# Use of Oclacitinib in the treatment of pemphigus foliaceus in a dog: case report

**DOI:** 10.29374/2527-2179.bjvm009024

**Published:** 2025-03-20

**Authors:** Millene Macieira Carneiro da Silva, Márcia Bernardini, Natália Lôres Lopes

**Affiliations:** 1 Universidade Iguaçu (UNIG), Nova Iguaçu, RJ, Brazil; 2 Autonomous veterinary, Rio de Janeiro, Brazil

**Keywords:** autoimmune disease, treatment, Janus kinase inhibitor, doença autoimune, tratamento, inibidor de Janus kinase

## Abstract

Pemphigus foliaceus is an autoimmune bullous dermatosis caused by the action of antibodies against epidermal cells and is considered as the most frequent pemphigus complex disease that affects pets. It is characterized by the presence of intraepidermal pustules that may evolve into erosion, scaling, crusts, and alopecia, affecting the periocular area, nasal planum, ventral area, abdomen, and paw pads. The diagnosis is made by observing the animal’s history, anamnesis, clinical signs, and laboratory tests such as cytological examination and is confirmed by histopathology. The recommended treatment is systemic immunosuppressive therapy, such as prednisone or prednisolone. This study reports the treatment of a male German Spitz canine with oral oclacitinib who was diagnosed with pemphigus foliaceus that was unresponsive to oral corticosteroids. Oclacitinib was effective in treating pemphigus foliaceus and may be a new therapeutic option for this dermatopathy.

## Resumo

O Pênfigo foliáceo é uma dermatose bolhosa autoimune, consequente da ação de anticorpos agindo contra as próprias células da epiderme, sendo conceituada como a mais frequente dentre as doenças do complexo pênfigo, que acomete os animais de companhia. É caracterizada pelo aparecimento de pústulas intraepidérmicas que podem evoluir para erosões, descamações, crostas e alopecia que afetam região periocular, plano nasal, região ventral, abdome e coxins. O diagnóstico, é obtido através da observação do histórico animal, anamnese, associado aos sinais clínicos e exames laboratoriais como o exame citológico e confirmado com o exame histopatológico. No tratamento é recomendado a terapia sistêmica, com a administração de imunossupressores como prednisona ou prednisolona. O presente estudo de caso, tem por objetivo relatar o tratamento com oclacitinib em um cão da raça Spitz Alemão, diagnosticado com pênfigo foliáceo arresponsivo ao tratamento com glicocorticoides. Dado ao exposto, o oclacitinib se mostrou eficaz quando utilizado para tratamento do pênfigo foliáceo, podendo ser uma nova opção terapêutica para essa dermatopatia.

## Introduction

Pemphigus foliaceus is an autoimmune disease (Medleau & Hlinica, 2003) characterized by acantholysis, which disrupts desmosomal junctions in the epidermis, leading to the formation of acantholytic cells (Miller et al., 2013). Clinical signs include the development of superficial pustules that evolve into crusts, epidermal collarettes, and erosion. Alopecia and hyperkeratosis can also observed. The most affected areas are the head, face, and ears, and nasal depigmentation may occur (Miller et al., 2013; Rhodes & Werner, 2014).

The diagnosis of pemphigus foliaceus is based on the history, clinical signs, and histopathology of intact pustules (Mencalha, 2019). Acantholytic cells, neutrophils, and eosinophils may be observed during cytopathological examinations (Raskin & Meyer, 2012). The histopathology of skin biopsies shows subcorneal pustules, acantholytic cells, and neutrophils, and eosinophils may also be observed (Mencalha, 2019; Miller et al., 2013)

The treatment of pemphigus foliaceus includes the administration of oral corticosteroids, such as prednisone or prednisolone (2 mg/kg). If this therapy fails to achieve efficacy in dogs, oral cicosporine could be considered as an option at 5-10 mg/kg once daily (Koch et al., 2012). Zhou et al. (2021) reported a mean remission time of 56 days. They observed that the most common adverse effects during treatment were diabetes, muscle wasting, weakness, and calcinosis cutis (Zhou et al., 2021).

Oclacitinib is a drug used to treat allergic diseases, such as atopic dermatitis, and is considered safe for long-term treatment (Cosgrove et al., 2013a; Simpson et al., 2017) and is fast and effective (Gonzales et al., 2014). Its mechanism of action is to control itching by neutralizing pruritogenic and pro-inflammatory cytokines and inhibiting Janus kinase (JAK) and, therefore, interleukin 31 (IL-31). Thus, oclacitinib prevents the animal from exhibiting signs of pruritus, as IL-31 levels are correlated with itching (Gonzales et al., 2014).

Recently, this drug has been used to treat autoimmune diseases (Levy et al., 2019). In a German Shepherd dog with subepidermal bullous dermatosis, a dose of 0.5 mg/kg twice a day demonstrated improvement in clinical signs after 1 month of treatment, and after 12 months no adverse effects were observed (Aymeric & Bensignor, 2017). In a cat with pemphigus foliaceus, oral oclacitinib was administered at a dose of 1 mg/kg twice daily, and after 1 week, a reduction in pruritus and severity of skin lesions was observed. After clinical improvement, the dose was reduced to 0.5 mg/kg twice daily (Carrasco et al., 2021).

This study aimed to report treatment with oclacitinib in a male German Spitz canine diagnosed with pemphigus foliaceus that was irresponsive to oral corticosteroid treatment.

## Case description

**Figure 1:**
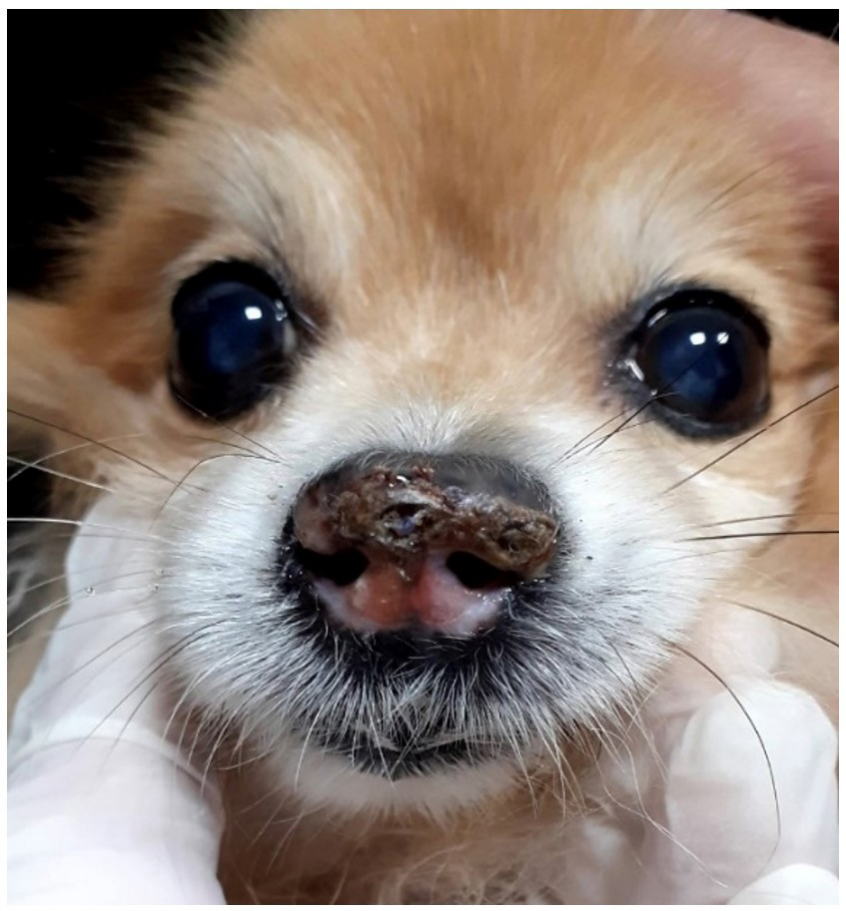
German Spitz dog, diagnosed with pemphigus foliaceus presenting depigmentatio, crusts and loss of nasal architecture in the nose.

A canine, male, German Spitz, 11 years old was attended to in a veterinary clinic in Rio de Janeiro because of nose depigmentation. The animal was treated with oral cyclosporine once a day and prednisolone every 48 h without a clinical response. Clinical examination revealed a loss of nasal mirror architecture, crusts in the nose, depigmentation, and ulceration (Figure 1). Blood test revealed thrombocytopenia, leukocytosis, lymphocytosis, and eosinophilia. Serum chemistry parameters (alanine transferase, alkaline phosphatase, urea, creatinine, triglycerides, cholesterol, total proteins, and fractions) were within the reference range. The patient had already undergone a histopathological examination before, which was inconclusive, sorology for leishmaniasis was negative, and 4DX and cytopathology without any alterations observed. Therefore, it was recommended to stop prednosolone administration to repeat the histopathological examination. A new bipsy was performed after 20 days without prednisolone, and it was characterized by the presence of orthokeratotic hyperkeratosis mixed with the formation of subcorneal pustules with segmented neutrophils and discrete aggregates of acantholytic cells, compatible with pemphigus foliaceus. Extensive foci of necrosis and epidermal ulcerations were observed in some sections.

**Figure 2:**
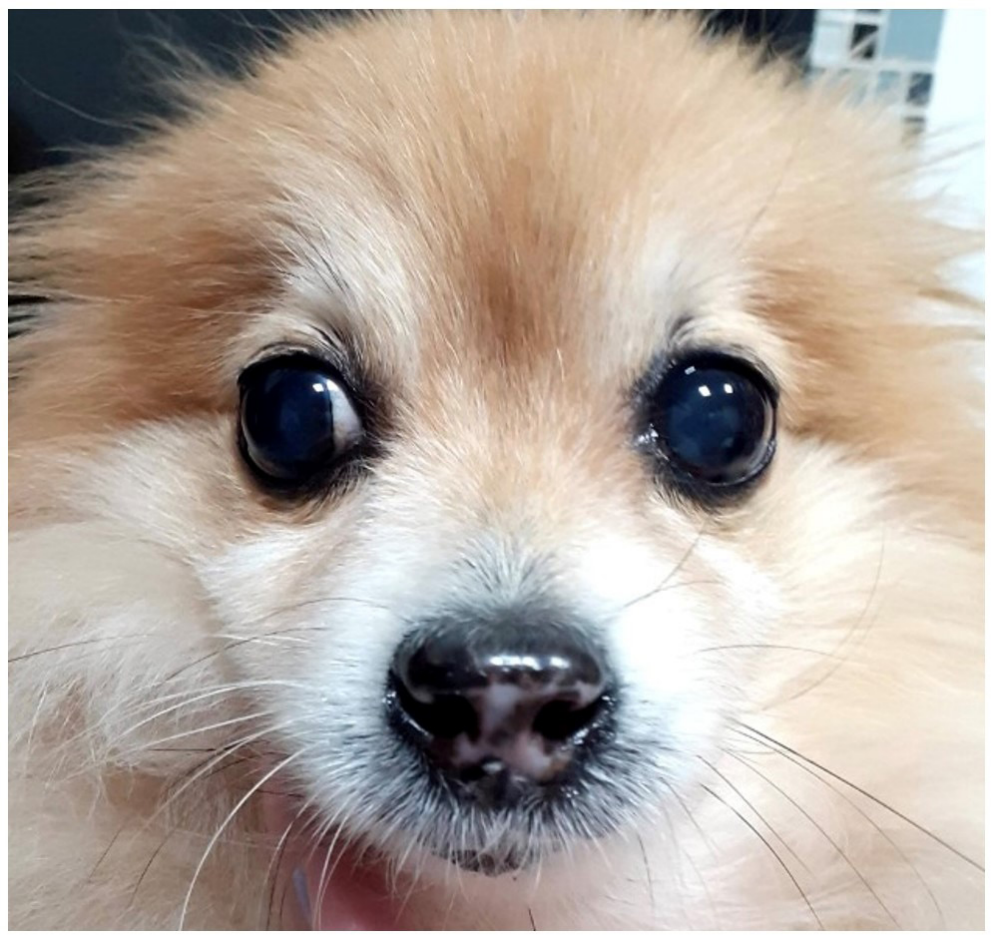
German Spitz dog, diagnosed with pemphigus foliaceus. After 14 days of treatment with oclacitinib nasal lesions healed, no crusts or ulcers observed.

**Figure 3:**
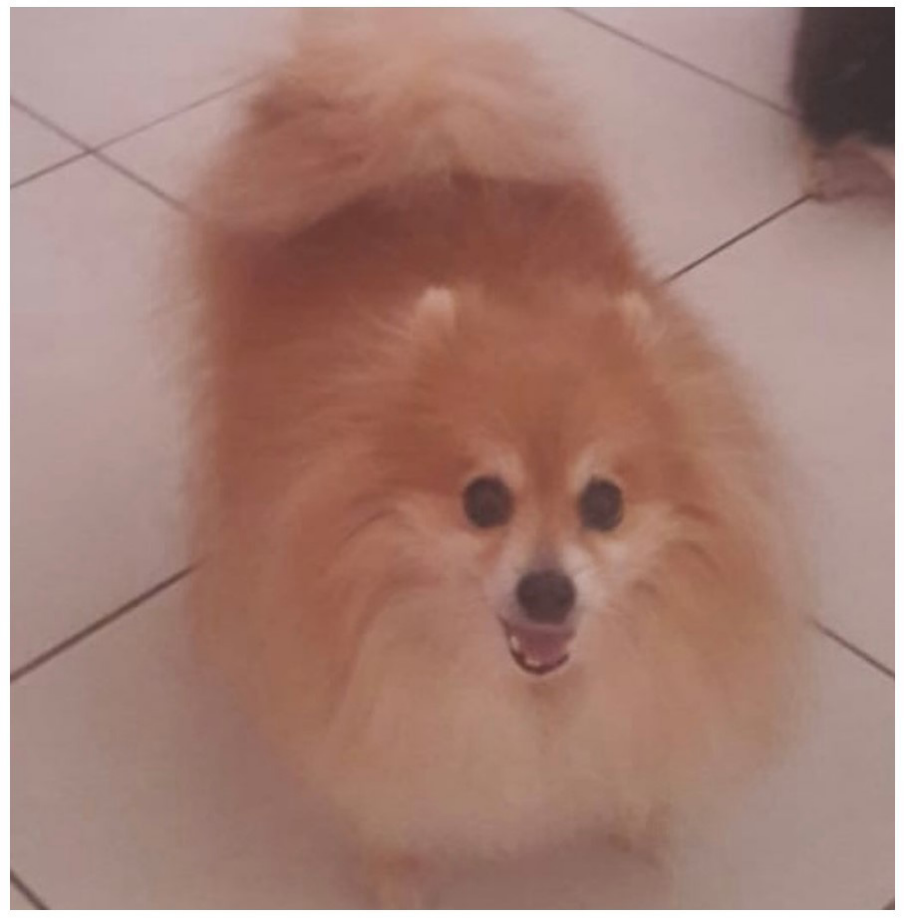
German Spitz dog, diagnosed with pemphigus foliaceus, presenting nasal lesions totally healed after treatment with oclacitinib.

As the dog was already receiving oral corticosteroids and cyclosporine for a while without any response, we decided to start treatment with oral oclacitinib 0.5 mg/kg twice a day for 14 days. After 14 days of treatment, improvement in the nasal lesions was observed, the ulcers healed (Figure 2), and the platelet count improved. Therefore, we decided to administer oclacitinib once a day. After 30 days of treatment, the nasal lesions completely healed and no depigmentation was observed (Figure 3). Routine follow-up tests were recommended. The patient did not experience any adverse effects during this period.

## Discussion

Pemphigus foliaceus is an autoimmune condition, and its pathogenesis involves the formation of autoantibodies that attack the patient´s own epidermal cells. Clinical signs include vesicles or pustules that adhere to form crusts, desquamation, erosion, erythema, hyperkeratosis, and ulceraion (Miller et al., 2013), as the skin lesions presented by the dog in this report. To obtain a definitive diagnosis, skin biopsy was performed, and intact fragments of the affected areas were collected for histopathological examination. This method is more suitable for obtaining good results by selecting an appropriate biopsy area, collecting samples from multiple regions, and preserving the surface of the lesion (Conceição, 2004). The association between clinical signs, animal history, and the examination is fundamental for the correct diagnosis (Barbosa, 2012).

The treatment of choice for pemphigus foliaceus is immunosuppressive therapy with corticosteroids (Medleau & Hlinica, 2003). In addition, immunosuppressive drugs such as cyclosporine and azatioprine may be used (Abreu et al., 2014; Alves et al., 2014). In this case, the patient was treated with corticosteroids and cyclosporine without a satisfactory response. Therefore, oral oclacitinib therapy was chosen as the treatment, which showed a good response in this case.

Therapy for autoimmune diseases may take an extended time and even life, and long-term treatment with glucocorticoids could lead to several adverse events in animals (Abreu et al., 2014; Pereira et al., 2011). However, oclacitinib has been reported to have a low frequency of adverse effects (Galati et al., 2018), and long-term use of this drug for atopic dermatitis has been shown to be safe and effective (Cosgrove et al., 2013b). These data may be useful to consider oclacitinib in patients for whom corticosteroid therapy is not an option, such as dogs with diabetes or hyperadrenocorticism, and to avoid adverse events associated with its use. It has also been demonstrated to be an effective glucocorticoid-sparing drug (Hernandez-Bures et al., 2023).

The mechanism of action of oclacitinib in animal autoimmune diseases has not yet been elucidated. Notably, JAK inhibitors have been used in humans to treat autoimmune diseases (Howell et al., 2019) with a satisfactory response (O’Shea et al., 2014; Tavakolour, 2018). Inhibition of JAK 1 and JAK 3 by this class of drugs has been observed in humans and animals, which subsequently inhibit interleukins (Gonzales et al., 2014; Juczynska et al., 2020). In humans diagnosed with pemphigus vulgaris, there is a greater expression of JAK 3 enzymes in skin lesions than in normal skin (Juczynska et al., 2020), which could explain why JAK inhibitors could be useful in treating these cases. The mechanism of action of oclacitinib involves inhibition of JAK 1 and 3 (Gonzales et al., 2014). This could explain why this drug worked well in the case reported here; however, more studies in dogs are needed to elucidate the involvement of JAKs in the pathogenesis of pemphigus foliaceus and the effectiveness of oclacitinib in this disease.

In animals, a few studies report the use of oclacitinib in autoimmune cases (Aymeric & Bensignor, 2017; Carrasco et al., 2021) like cutaneous lupus erythematosus with doses varying from 0.45 mg/kg twice daily to 1.8 mh/kg once daily (Harvey et al., 2023). The patient in this report showed a good response at a dose of 0.5 mg/kg and, in contrast to other reports (Aymeric & Bensignor, 2017), and even when the administration interval was reduced to once a day, the dog continued to respond to thetreatment. In addition, the patient did not experience any adverse effects after oral oclacitinib administration, corroborating the data available in the literature showing that oclacitinib has a low incidence of adverse events. Similar to this report, a a dog diagnosed with cutaneous lupus erythematosus was successfully treated with oral oclacitinib twice a day for 15 days and then once a day at a dose 0f 0.6 mg/kg (Lima & Cunha, 2022).

## Conclusion

Oral oclacitinib was safe and effective for the treatment of pemphigus foliaceus in a dog, without any side effects. Therefore, this drug may serve as a new treatment option for this disease. More studies with a larger number of patients are needed to evaluate its long-term efficacy.
